# The psychological factors mediating/moderating the association between body‐image disturbance and depression: A systematic review

**DOI:** 10.1002/pchj.754

**Published:** 2024-05-10

**Authors:** Yiyi Wang, Yuqi Chen, Chenxuan Lu, Angela T. H. Kwan, Roger S. McIntyre, Fahui Yang, Bing Cao

**Affiliations:** ^1^ Key Laboratory of Cognition and Personality, Faculty of Psychology, Ministry of Education Southwest University Chongqing China; ^2^ Brain and Cognition Discovery Foundation Toronto Ontario Canada; ^3^ Faculty of Medicine University of Ottawa Ottawa Ontario Canada; ^4^ Department of Pharmacology and Toxicology University of Toronto Toronto Ontario Canada; ^5^ Department of Psychiatry University of Toronto Toronto Ontario Canada; ^6^ National Demonstration Center for Experimental Psychology Education Southwest University Chongqing China

**Keywords:** body‐image disturbance, depression, perceived stress, psychological mechanism, self‐esteem, social support

## Abstract

Available evidence demonstrates that individuals with body‐image disturbance (BID) are prone to suffer from depression. This systematic review provides, to our knowledge, the first synthesis of the psychological mechanism of the association between BID and depression. We conducted a thorough search of online databases, including PubMed, Web of Science, and PsycINFO, for articles published up until February 2024. The final analysis comprised a total of 23 studies that focused on the mediating or moderating effects of psychological factors between depression and BID. This review identifies self‐esteem and social support as both mediators and moderators of the relationship between BID and depression, while perceived stress acted only as a mediator. High self‐esteem and strong social support as well as low levels of perceived stress may help individuals experience lower levels of BID, thereby contributing to a decreased likelihood of depression. Interventions aimed at increasing self‐esteem, developing strong support, and decreasing perceived stress may hold promise to reduce the risk of depression in those with BID.

## INTRODUCTION

Body‐image disturbance (BID) is defined as a distortion of perception, behavior, or cognition due to weight or shape (Pimenta et al., 2009). BID also refers to the discrepancy between perceived and ideal body image (Ferrer‐Garcia & Gutierrez‐Maldonado, 2012), which has been negatively associated with quality of life to a greater extent than body mass index (BMI) [kg/m^2^] (Wilson et al., 2013). A body of studies has found that BID has a detrimental impact on psychological functioning (Wilson et al., 2013) and it has been recognized as a predictor of depression (Gardner & Brown, 2014; Paans et al., 2018) and bulimia (Keel et al., 2001).

BID and depression have been proved to be strongly correlated in numerous studies. A cross‐sectional study revealed that BID was associated with depression regardless of BMI, sex, and age (Richard et al., 2016). A cross‐lagged panel study considering the bidirectional relationships between body dissatisfaction and depressive symptoms suggested that the prevention of body dissatisfaction and depressive symptoms may be mutually beneficial and potentially combined (Wang et al., 2020). Two potential hypotheses have been proposed to explain the association of body image and depression. One is that depression is related to negative self‐appraisal, and BID is a manifestation of negative self‐appraisal that results from depression (Cohentovee, 1993; Cooper & Taylor, 1988). Body dissatisfaction, on the other hand, is likely to lead to stricter diets, which may contribute to depression because emotional distress may arise as a result of dietary failures or as a direct result of the negative effects of calorie restriction on mood (Stice & Bearman, 2001). However, the direction of causation between BID and depression remains unknown, as the majority of existing studies are cross‐sectional, including both BID as an independent variable and depression as an independent variable, making it difficult to determine a bidirectional link between BID and depression. More original longitudinal studies are needed in the future to investigate their specific mechanisms. Given the foregoing, our study focused on the mechanism leading from BID to depression. In the future, we will also pay more attention to the potential causal mechanisms between BID and depression.

Despite the growing evidence that BID may lead to increased levels of depression, a mediation or moderation model linking BID and depression has not, to our knowledge, been systematically defined. Exploring mediators and moderators between these two conditions is critical in clinical prevention, as interventions can specifically target these factors to help reduce BID‐related depressive symptoms (Ma et al., 2022). Many studies have investigated the potential mediators between BID and depression, which include self‐regard psychological qualities, such as self‐esteem, self‐compassion, and self‐efficacy (Brechan & Kvalem, 2015; Pehlivan et al., 2022; Przezdziecki et al., 2013; Todd, 2007), as well as interpersonal relationship such as social connectedness (McGregor, 2022), and perceived stress (Sabik et al., 2019; Ziser et al., 2019). Furthermore, potential moderators influencing the direction or degree of connection between BID and depression have also been found, with some of them also demonstrating a major mediating function, as mentioned above. These moderators include self‐compassion (Sick et al., 2020), parental attachment security (Hoffman, 2007), and sense of belonging (Hanley & McLaren, 2015).

Therefore, there is a need to integrate the vast and varied literature to examine whether these psychological factors consistently play a significant role in the association between BID and depression. Accordingly, the purpose of this review is to systematically summarize the psychological mechanism leading from BID to depression in the existing literature.

## METHODS

### Search strategy

A systematic review was conducted according to the Preferred Reporting Items for Systematic Reviews and Meta‐Analyses (PRISMA) 2020 (Page et al., 2021), and the PRISMA checklist is provided in Appendix S1. Systematic searches of articles in PubMed, Web of Science, PsycINFO, China National Knowledge infrastructure (CNKi), Wanfang Academic Journal Full‐text Database (Wanfang), Chongqing VIP Database (CQVIP) were conducted up to February 2024, using a combination of controlled terms and keywords. The searching strategy was: (depression OR depress* OR depressive OR MDD OR bipolar disorder) AND (body image disturbance OR body dissatisfaction OR body shame OR body preoccupation OR body attitudes OR body image OR body image concern OR body image distortion OR weight concern). The detailed search strategies in the database are displayed in Appendix S2. Only original studies involving human subjects and written in English or Chinese were included in the searches. In addition, the reference lists of related reviews and included articles were manually searched to ensure that as many relevant studies as possible were considered for inclusion.

All relevant articles retrieved from the database searches were exported to Rayyan Qatar Computing Research Institute (QCRI, https://rayyan.qcri.org) after the removal of duplicates (Ouzzani et al., 2016). To determine eligibility in accordance with the inclusion criteria established for this review, two reviewers (WYY and CYQ) went through the titles and abstracts independently. For all studies that met (or potentially met) the inclusion criteria, the full article was retrieved. The review protocol has been registered with the International Prospective Register of Systematic Reviews PROSPERO (CRD42023388094).

### Identification of eligible studies

The inclusion criteria were: (1) depressive outcomes should be clinically diagnosed or measured by a validated tool (e.g., Center of Epidemiological Studies Depression Scale (CES‐D), Patient Health Questionnaire‐9 (PHQ‐9)); (2) the research pathway in the study should be predicting depression from BID; (3) the variables between BID and depression are psychological variables. The exclusion criteria were: (1) animal studies; (2) targets of biological or demographic variables between BID and depression (e.g., gender, BMI, age, country); (3) the variables between BID and depression are biological or demographic variables; (4) no access to the full text.

### Data extraction

Data were extracted and synthesized in an Excel file in the following format: (1) article information; (2) demographic features of participants; (3) study design type; (4) measurement for BID, depression, and mediators/moderators; (5) key findings about the psychological mechanism leading from BID to depression (only relevant results were reported).

### Quality assessment

The Joanna Briggs Institute (JBI) Critical Appraisal Checklist for Analytical Cross‐Sectional Research was used to evaluate the quality of cross‐sectional research. The JBI Critical Appraisal Checklist for Cohort Studies was used to evaluate the longitudinal studies (Moola et al., 2015; Santos et al., 2018; Zhao et al., 2022).

## RESULTS

### Search results

**FIGURE 1:**
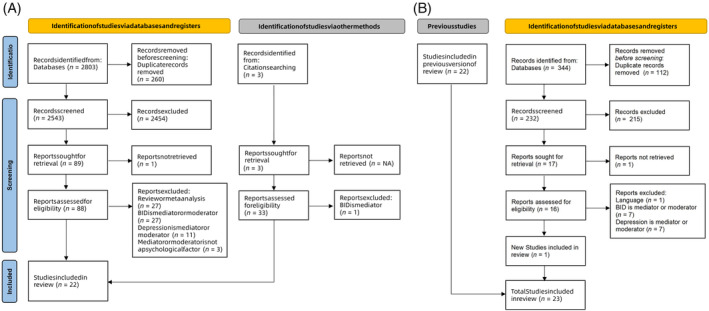
Flowchart of the study selection.

**FIGURE 2:**
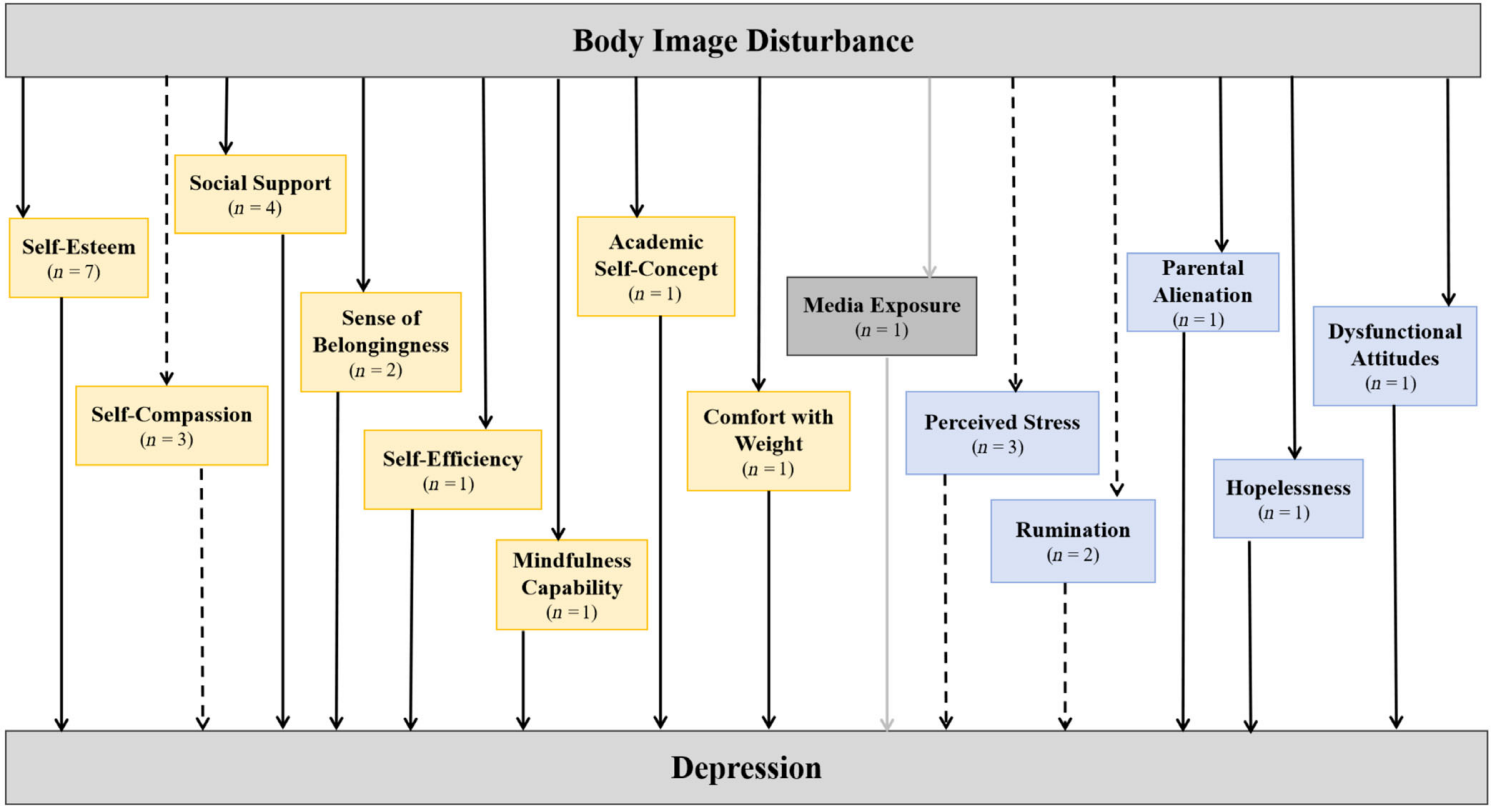
Variables associated with the relationship between body image disturbance (BID) and depression. Key: yellow block = protecting mediators/moderators; blue block = deteriorating mediators/moderators; gray block = insignificant mediators/moderators; solid line = significant association of the relationship in all the included studies; dotted line = significant association of the relationship in some of the included studies; gray line = no significant association of the relationship in any of the included studies; *n* = # is the number of studies examining the factor.

The full overview of the study selection procedure is depicted in Figure 1. After two rounds of initial search, a total of 3147 studies were identified in the online database. After removing 318 duplicate search results, 2829 articles were examined for eligibility. A total of 21 full‐text articles met our inclusion requirements. To guarantee a more thorough search, we also manually checked the reference lists of relevant reviews and included articles. A further two studies were eventually included. In total, there were 20 cross‐sectional studies and three longitudinal studies in our review (Table 1). Among these 23 studies, 14 psychological factors were evaluated. A summary of the associations between all variables in the literature reviewed can be found in Figure 2.

**TABLE 1:** Summary of studies included in final review.

Author, year	Country	Sample characteristics	Study design	Body‐image assessment	Depression assessment	Mediator/moderator assessment	Key findings
Brechan & Kvalem, 2015	Norway	320 college students, 65% female, Mage = 24.26	Cross‐sectional	BIQ	BDI‐II	Self‐esteem, RSES	Significant indirect effect of body dissatisfaction on depression through self‐esteem (*p* < .001).
Brewis & Bruening, 2018	United States	534 college students, 71.5% female	Longitudinal	1 item from Vartanian and Shaprow (2008)	4 items from American College Health Association validated protocols (2013)	Social connection, 10 items from Buote et al. (2007), Bui (2002), and McAndrew (1998)	Social connections mediate earlier in the school year (indirect effect = 0.06), and in all phases moderate body‐shame effects on depression (*p* < .001).
Brown, 2008	United States	101 female teachers, Mage = 41.13	Cross‐sectional	MBSRQ‐AS	CES‐D	Self‐compassion, SCS	Self‐compassion did not moderate the relationship between body‐image satisfaction and depression (*p* > .05).
Brunet et al., 2019	Canada	811 young adults, 55.2% female, Mage = 24.02	Cross‐sectional	WEB‐SG	MDI	Self‐esteem, RSES	Self‐esteem moderated the association between body‐related guilt and depressive symptoms (*β* = .33, 95% CI = [.39, 5.14]).
Choi & Choi, 2016	South Korea	1002 American adolescents, 54.9% female, 3993 Korean adolescents, 48.3% female	Cross‐sectional	4‐point scale ranging from very satisfied to not satisfied at all	3 items developed by Choi et al. (2010)	Self‐esteem, 5 items developed by Choi et al. (2010)	The indirect effect of BID on depression via self‐esteem was greater for American adolescents (*b* = .21, 95% CI = [.17, .25]), than for Korean adolescents (*b* = .05, 95% CI = [.02, .12]).
Duan and Wang, 2019	China	293 overweight or obese college students, 52.9% female, Mage = 22.5	Cross‐sectional	4‐item scale measure of WSC (Hunger & Major, 2015)	DASS‐21	Mindfulness capability, SIM‐C	The results verify the significant indirect effects of weight‐based stigma on negative emotion through mindfulness capability (effect = 0.0160, 95% CI = [.0059, .0302])
Duchesne et al., 2017	Canada	409 adolescents, 58.4% female, Mage = 15.7	Cross‐sectional	Series of figures developed by Thompson and Gray (1995)	CES‐D	Self‐esteem, RSES	The indirect effects were significant for depression (effect = −1.30; SD = .28; 95% CI = [−1.88, −.78]), which confirmed the mediating role of self‐esteem
Evans, 2011	United States	269 female undergraduate students, Mage = 22	Cross‐sectional	OBCS	HDSQ	Hopelessness, Hopelessness Scale	Hopelessness partially mediated the relationship between body shame and depression (*p* < .001).
Ferreiro et al., 2012	Spain	376 girls (Mage = 14.98) and 372 boys (Mage = 14.99)	Longitudinal	The Body Dissatisfaction subscale of EDI‐2	CDI	Social support, 3 items by the author	For boys only, social support moderated the effect of BID on depressive symptoms (*β* = −.61, *p* < .05)
Hanley & McLaren, 2015	Australia	162 self‐identified Australian lesbians, Mage = 38.19	Cross‐sectional	BSS	CES‐D	Sense of belonging, psychological subscale of SOBI	Sense of belonging to each of 3 layers of the lesbian community: abroad (*β* = −.04, *p* < .001), organizational (*β* = −.10, *p* < .001), and friendship (*β* = −.15, *p* < .001) moderated the BID→depressive symptoms relationship
Hassani et al., 2021	Iran	165 patients with psoriasis, 29.6% female, Mage = 36.62	Cross‐sectional	BIS; BISQ	SCL‐90	Self‐esteem, RSES	Significant indirect effect of body dissatisfaction on depression through self‐esteem in men and women (*p* < .05)
Hoffman, 2007	United States	89 high school students, 69% female, Mage = 14.7	Cross‐sectional	BSRQ‐AES	CES‐D; POMS	Parental attachment security, IPPA; Media exposure, daily diary	Overall parental attachment security did not moderate the relationship between BID and depressive symptoms (*p* = .33), but parental alienation was a significant moderator (*p* < .001). Media exposure was not significantly related to BID or depressed mood (*p* = .31)
Koronczai et al., 2013	Hungary	694 participants between 14 and 34 years, 41.5% female, Mage = 21.5	Cross‐sectional	8‐item questionnaire designed by the authors	CES‐D	Self‐esteem, RSES	The satisfaction with body appearance→self‐esteem→depression→problematic internet use pathway appeared to be significant (*p* < .001 for males, *p* < .001 for females)
Liu et al., 2017	Singapore	221 adult cancer patients, 67.4% female, Mage = 48.2	Cross‐sectional	BIS	HADS	Dysfunctional attitudes, DAS; Rumination, RRS	Both dysfunctional attitudes (*b* = .04, *p* = .01) and rumination (*b* = .29, *p* = .001) mediated the relationship between BIC and emotional distress
McGregor, 2022	United States	151 participants, male‐identifying undergraduate students, Mage = 21.3	Cross‐sectional	BAS‐2; MEBBIE‐2; BES‐R	BDI‐II; CESD‐R; HSCL‐58	Academic self‐concept, Self‐Description Questionnaire III; Social connectedness, SCS‐R	Academic self‐concept significantly moderated the relationship between body image and depressive symptoms (*b* = −.03, *p* = .03). Social connectedness significantly moderated the relationship between BID and depressive symptoms (*b* = −.01, *p* = .02)
Morken et al., 2019	Norway	547 adolescents, 55% female, Mage = 12.58	Cross‐sectional	SPPA‐R	SMFQ	Social support from peers, Social Support Scale; Social support from parents, The Parental Warmth/Involvement subscale	Peer support moderated the association between body image and depressive symptoms (*β* = −.09, *p* = .021). Parental support did not moderate the relationship between body image and depressive symptoms (*β* = −.03, *p* = .48)
Pehlivan et al., 2022	Australia	451 patients with endometriosis, over 18 years of age	Longitudinal	BIS	DASS‐21	Self‐esteem, RSES; Rumination, MRIS	Significant indirect effect of body image on depression at T2 through self‐esteem at T1 (a1 b1 = .04, 95% CI = [.02, .09]). Rumination at T1 did not significantly mediate the body‐image depression pathway (a2 b2 < .01, 95% CI = [−.01, .02]). Poorer body image at T0 was only marginally associated with greater rumination at T1 (a2‐path = .06, 95% CI = [−.01, .12]) and greater rumination at T1 was not predictive of higher depression at T2 (b2‐path = .07, 95% CI = [−.06, .20]).
Przezdziecki et al., 2013	Australia	279 women with breast cancer, over 18 years of age	Cross‐sectional	BIS	DASS‐21	Self‐compassion, SCS; Pressure from others, 4 Yes/No‐format items; Comfort with weight, 3‐item scale	For depression, comfort with weight (*β* = .07, 95% CI = [.00, .14]) met the criteria for mediation, along with self‐compassion (*β* = .22, 95% CI = [.14, .33]). Pressure from others did not meet the criteria for mediator (β = .03, 95% CI = [−.02, .09])
Roberts, 2023	United States	75 youths with inflammatory bowel disease between 10 and 18 years and their parent or legal guardian	Cross‐sectional	YBIQ	CDI‐2	Thwarted belongingness, INQ‐TB	Analyses revealed a significant serial indirect path for illness stigma→body‐image dissatisfaction→thwarted belongingness→depressive symptoms (effect = .81, 95% CI = [.15, 1.78])
Sabik et al., 2019	United States	57 undergraduates, 49.1% female, Mage = 20.13	Cross‐sectional	BES	CES‐D	Stress, TSST, PSS, salivary cortisol measurement	Chronic stress mediated the association between body image and depressive symptoms (*b* = 1.09; 95% CI = [−7.00, −1.32])
Sick et al., 2020	Canada	520 adults, 42.3% female, Mage = 35.43	Cross‐sectional	BF‐SGS	BDI‐II	Self‐compassion, SCS‐SF	Self‐compassion moderated the relationship between body‐related shame and depression among women (∆*R* ^2^ = 0.01, *p* = .009), but not among men, even after controlling for self‐esteem (∆*R* ^2^ < 0.01, *p* = .202)
Todd, 2007	United States	198 females, Mage = 28.39	Cross‐sectional	OBCS	CES‐D	General self‐efficacy, SES; Physical self‐efficacy, PSE	General self‐efficacy mediated the relationship between body shame (*β* = −.258, *p* < .001), body surveillance (*β* = −.286, *p* < .001), and symptoms of depression. Physical self‐efficacy mediated the relationship between body surveillance and symptoms of depression (*β* = .197, *p* < .001)
Ziser et al., 2019	Germany	579 patients with obesity (BMI ≥30 kg/m^2^), 70.3% female, Mage = 41.68	Cross‐sectional	BIQ‐20	PHQ‐9	Perceived stress, PSQ‐20	Perceived stress partially mediated NEB (*b* = .09, *p* < .001) as well as PBD (b = −.21, *p* < .001) and depression.

Abbreviations: BAS‐2 = Body Appreciation Scale, 2nd version; BDI = Beck Depression Inventory; BES = Body‐Esteem Scale; BF‐SGS = scenario‐based self‐report Body‐Focused Shame and Guilt Scale; BIC = body image concern; BIQ = Body Image Questionnaire; BIS = Body Image Scale; BISQ = Body Image Satisfaction Questionnaire; BSRQ‐AES = Body–Self Relations Questionnaire: Appearance Evaluation Subscale; BSS = Body Satisfaction Scale; CDI = Children's Depression Inventory; CES‐D = Center of Epidemiological Studies Depression Scale; DAS = Dysfunctional Attitudes Scale; DASS = Depression Anxiety and Stress Scale; EDI‐2 = Eating Disorder Inventory‐2; HADS = Hospital Anxiety and Depression Scale; HDSQ = Hopelessness Depression Symptoms Questionnaire; HSCL‐58 = Hopkins Symptom Checklist; INQ‐TB = Interpersonal Needs Questionnaire‐Thwarted Belongingness Subscale; IPPA = Inventory of Parent and Peer Attachment; MBSRQ‐AS = Multidimensional Body Self‐Relations Questionnaire‐Appearance Scales; MDI = Major Depression Inventory; MEBBIE‐2 = Male Eating Behavior and Body Image‐Evaluation‐2; MRIS = Multi‐Dimensional Rumination in Illness Scale; NEB = negative evaluation of the body; OBCS = Objectified Body Consciousness Scale; PBD = positive body dynamics; PHQ = Patient Health Questionnaire; POMS = profile of mood states; PSE = Physical Self‐Efficacy Scale; PSQ = Perceived Stress Questionnaire; PSS = perceived chronic stress; RRS = Ruminative Response Scale; RSES = Rosenberg Self‐Esteem Scale; SCL‐90 = Symptom Checklist 90; SCS = Self‐Compassion Scale; SCS‐R = Social Connectedness Scale‐Revised; SES = Self‐Efficacy Scale; SIM‐C = Short Inventory of Mindfulness Capability; SMFQ = Short Mood and Feeling Questionnaire; SOBI = Sense of Belonging Instrument; SPPA‐R = Self‐Perception Profile for Adolescents; TSST = Trier Social Stress Test; WEB‐SG = Weight and Body‐Related Shame and Guilt; WSC = weight stigma concern; YBIQ = Youth Body Image Questionnaire.

A detailed description of the study characteristics is provided in Table 1. With sample sizes ranging from 57 to 1179, the majority of studies (*n* = 16, 69.57%) were published after 2015 and were mostly in North America (*n* = 11, 47.82%) and Europe (*n* = 5, 21.74%). The populations varied among these studies. There were five studies focusing on children or adolescents, five studies on college students, and five studies on adults. A further seven studies were aimed at individuals with a diagnosed disease (e.g., obesity, endometriosis, inflammatory bowel disease, psoriasis, cancer). The remaining study assessed lesbians in LGBT groups.

#### Checklist for quality

The quality assessment follows the JBI Critical Assessment Checklist, as detailed in Appendix S3. Overall, all included studies met the quality assessment criteria, but some studies suffered from some quality assessment problems. There were 10 cross‐sectional studies that did not clearly define the criteria needed for inclusion and a further 10 cross‐sectional studies that did not identify and deal with the confounding factors. As for the three longitudinal studies, none of them disclosed the methods used to deal with incomplete follow‐up. Furthermore, one of them was of lower quality owing to its inability to identify confounding variables.

### Predicting depression from body‐image disturbance

The different studies considered in this review assessed BID in different ways. Fifteen studies used a valid and reliable existing scale, such as the Body Image Scale (BIS; *n* = 4; Hassani et al., 2021; Liu et al., 2017; Pehlivan et al., 2022; Przezdziecki et al., 2013), Body Image Questionnaire (BIQ; *n* = 3; Brechan & Kvalem, 2015; Roberts, 2023; Ziser et al., 2019), Objectified Body Consciousness Scale (OBCS; *n* = 2; Evans, 2011; Todd, 2007). The Body‐Esteem Scale (BES; Sabik et al., 2019), Multidimensional Body Self‐Relations Questionnaire (MBSRQ; Brown, 2008), Weight and Body‐Related Shame and Guilt (WEB‐SG; Brunet et al., 2019), Body Dissatisfaction subscale of Eating Disorder Inventory (EDI; Ferreiro et al., 2012), Body Satisfaction Scale (BSS; Hanley & McLaren, 2015), Body–Self Relations Questionnaire (BSRQ; Hoffman, 2007), Self‐Perception Profile for Adolescents (SPPA; Morken et al., 2019), and Scenario‐Based Self‐Report Body‐Focused Shame and Guilt (BF‐SGS; Sick et al., 2020) were each used once to assess BID. One of the studies used the BES, Body Appreciation Scale (BAS), and Male Eating Behavior and Body Image‐Evaluation (MEBBIE) together. Moreover, four studies applied item scales (Brewis & Bruening, 2018; Choi & Choi, 2016; Duan & Wang, 2019; Koronczai et al., 2013), while one study assessed BID using a visual graph table (Duchesne et al., 2017).

In terms of assessing depression, the main scale used was the Center for Epidemiological Studies Depression Scale (CES‐D; *n* = 6). The Beck Depression Inventory (BDI), Children's Depression Inventory (CDI), Depression Anxiety and Stress Scale (DASS) were each used three times for the measurement of depression. And the following scales were only used once in the included studies: Patient Health Questionnaire (PHQ‐9), Major Depression Inventory (MDI), Hopelessness Depression Symptoms Questionnaire (HDSQ), Short Mood and Feeling Questionnaire (SMFQ), Hospital Anxiety and Depression Scale (HADS), Symptom Checklist 90 (SCL‐90). Two studies used more than one scale to evaluate depressive symptoms, one with the combination of CES‐D and Profile of Mood States (POMS), and the other with BDI‐II, CES‐D, and Hopkins Symptom Checklist (HSCL) together. The remaining two studies utilized item scales from the American College Health Association validated protocols that were developed by Injae Choi (2013).

The majority of the included studies discovered a significant effect of association between BID and depression; however, there were a handful that did not. There were 21 studies that detected significant associations between BID and depressive outcomes. Eighteen cross‐sectional studies found a positive correlation between body‐image‐related factors and depressive symptoms (Brechan & Kvalem, 2015; Brunet et al., 2019; Duan & Wang, 2019; Duchesne et al., 2017; Evans, 2011; Hanley & McLaren, 2015; Hassani et al., 2021; Hoffman, 2007; Koronczai et al., 2013; Liu et al., 2017; McGregor, 2022; Morken et al., 2019; Przezdziecki et al., 2013; Roberts, 2023; Sabik et al., 2019; Sick et al., 2020; Todd, 2007; Ziser et al., 2019), and three longitudinal studies found that BID directly and consistently predicted depression throughout all phases (Brewis & Bruening, 2018; Ferreiro et al., 2012; Pehlivan et al., 2022). Only two studies found no significant association between body dissatisfaction and depression, with one finding that body dissatisfaction was not related to depressive symptoms among female teachers (Brown, 2008) and the other only revealing an indirect effect of body dissatisfaction on depressive symptoms via self‐esteem (Choi & Choi, 2016).

### Protecting mediators/moderators

#### Self‐esteem

According to Rosenberg, self‐esteem refers to the value and respect that people attribute to themselves, which has been associated with body dissatisfaction and depression in many studies (Rosenberg, 1979). The psychological mechanism of self‐esteem was explored in seven studies in this review (Brechan & Kvalem, 2015; Brunet et al., 2019; Choi & Choi, 2016; Duchesne et al., 2017; Hassani et al., 2021; Koronczai et al., 2013; Pehlivan et al., 2022). Six studies applied the Rosenberg Self‐Esteem Scale (RESE) to assess self‐esteem, while the remaining study used a 5‐item scale (Choi & Choi, 2016). All of these studies demonstrated good overall quality, although several items did not meet the JBI criteria. Except for Hassani et al.'s study (2021) , five cross‐sectional studies did not clarify the inclusion criteria. Brechan and Kvalem (2015), Choi and Choi (2016), Hassani et al. (2021), and Koronczai et al. (2013) failed to identify and address confounding variables, while Choi and Choi (2016) failed to specify the condition's measurement criteria. The longitudinal study of Pehlivan did not report the strategies used to cope with incomplete follow‐up.

The mediating role of self‐esteem was found in five studies, with BID predicting symptoms of depression through association with self‐esteem (Brechan & Kvalem, 2015; Choi & Choi, 2016; Hassani et al., 2021; Koronczai et al., 2013; Pehlivan et al., 2022). Thus, a negative perception of one's body image  lowered self‐esteem, which in turn had an effect on psychological distress. Furthermore, two studies demonstrated that self‐esteem acted as a moderator, reducing the impact of BID on depressed outcomes (Brunet et al., 2019; Duchesne et al., 2017). It indicated that, in comparison to those with higher self‐esteem, those with lower self‐esteem had a stronger correlation between BID and depressive symptoms. Taken together, self‐esteem either can be impaired by BID, which then influences depression, or has a buffering effect on the impact of BID and depressive symptoms.

#### Self‐compassion

Self‐compassion is a prominent source of positive self‐regard, which involves being compassionate to oneself, acknowledging one's common humanity, and being mindful when evaluating bad parts of oneself (Neff, 2011). Three studies considered the influence of self‐compassion, all of which used the Self‐Compassion Scale (SCS; Brown, 2008; Przezdziecki et al., 2013; Sick et al., 2020). Regarding the evaluation of study quality, Brown's study (2008) suffered from poor quality because the inclusion criteria were not clearly defined, and neither Brown, 2008 nor Przezdziecki et al. (2013) identified or dealt with the confounding factors; however, Sick et al.'s study (2020) satisfied all the requirements and demonstrated excellent quality.

A mediating role of self‐compassion was discovered in Przezdziecki et al.'s study among breast cancer survivors, which showed that for depression, self‐compassion met the criteria for mediation (Przezdziecki et al., 2013). Regarding the moderating role, Sick discovered that, even after controlling for self‐esteem, self‐compassion moderated the connection between body‐related guilt and depression in female adults but not in male adults (Sick et al., 2020). However, the remaining study, focusing on female teachers, found no significant moderating role of self‐compassion (Brown, 2008). It remains unclear whether self‐compassion is the only a significant moderator in both male and female groups.

#### Social support and social connection

Social support refers to the provision of material and interpersonal resources by significant others in times of need (Kok et al., 2023). There were four studies examining the role of social support and social connection between BID and depression (Brewis & Bruening, 2018; Ferreiro et al., 2012; McGregor, 2022; Morken et al., 2019). The studies used several scales to evaluate social support, with two using existing scales, namely the Social Connectedness Scale (SCS; McGregor, 2022) and a combination of the Social Support Scale and the Parental Warmth/Involvement Subscale (Morken et al., 2019), and the other two using item scales (Brewis & Bruening, 2018; Ferreiro et al., 2012). With reference to the JBI checklist, the longitudinal studies by Brewis and Bruening (2018) and Ferreiro et al. (2012) did not address the use of incomplete follow‐up, and, furthermore, the participants were not free of the outcome at the start of the study. McGregor's (2022) cross‐sectional study satisfied all the quality requirements, but Morken et al.'s (2019) lacked a clear definition of the inclusion criteria.

In the cross‐sectional study targeting male‐identifying undergraduate students, social connection was evaluated as a potential mediator, indicating a strong indirect link between body dissatisfaction and depressed symptoms (McGregor, 2022). Furthermore, social support was found to operate as a moderator in two studies. Based on the findings of a longitudinal study aimed at adolescents, social support moderated the influence of body image on depressive symptoms only in young males (Ferreiro et al., 2012), while another cross‐sectional study of adolescents discovered that peer support but not parent support moderated the relationship between body image and depressive symptoms (Morken et al., 2019). Only one longitudinal study among college students examined not only the mediator role of social connection but also the moderator role, revealing that social connection mediated earlier in the school year and moderated BID effects on depression in all periods (Brewis & Bruening, 2018). It is possible to deduce that feeling more socially connected may reduce the risk of body‐shame‐related depressive symptoms.

#### Sense of belongingness

A sense of belongingness has been regarded as both an essential component of human functioning and a fundamental human motivator (Baumeister & Leary, 1995). Two studies explored the impact of a sense of belonging. The protective impact of belongingness, as assessed by the psychological subscale of the Sense of Belonging Instrument (SOBI), was demonstrated in cross‐sectional research among self‐identified Australian lesbians. It was discovered that the association between BID and depressive symptoms was moderated by a sense of belonging to each of the three layers of the lesbian community: organizational, friendship, and overseas (Hanley & McLaren, 2015). Additionally, a cross‐sectional study aimed at young people with inflammatory bowel disease (IBD) found a significant serial indirect path for illness stigma→body image dissatisfaction→thwarted belongingness→depressive symptoms (Roberts, 2023). The Interpersonal Needs Questionnaire‐Thwarted Belongingness subscale (INQ‐TB) was utilized to assess belongingness. It suggested that youths who perceived higher IBD stigma were more likely to have increased body‐image dissatisfaction, which could lead to feelings of social estrangement and, eventually, to elevated depressive symptoms. To summarize, the protective effect of belongingness and the deteriorating effect of thwarted belongingness may be combined to reach the consistent conclusion that the sense of belongingness could protect individuals with BID from depression.

#### Self‐efficiency

Bandura (1986) defined self‐efficiency as perceptions that an individual has about their ability to produce the needed levels of performance to influence events that affect their lives. Only one cross‐sectional study of female participants looked at the role of self‐efficacy in BID and depression, dividing it into two dimensions: general self‐efficacy and physical self‐efficacy (Todd, 2007). The former was evaluated using the Self‐Efficacy Scale (SES), while the latter was evaluated using the Physical Self‐Efficacy Scale (PSE). Except for the clear inclusion, all of the quality criteria were met. It was found that the association between body shame, body surveillance, and depressive symptoms was mediated by general self‐efficacy. Furthermore, the association between body surveillance and depressive symptoms was mediated by physical self‐efficacy.

#### Mindfulness capability

Mindfulness is defined as balancing one's mental resources against perceived pressures (Garland et al., 2009). Mindfulness capability involves both present‐centered awareness and a non‐judgmental attitude that allows individuals to mitigate the impact of weight stigma by paying intentional attention to external and internal experiences and maintaining a non‐evaluative attitude, making it a promising resiliency mechanism against negative emotions (Duan & Li, 2016). A cross‐sectional study of overweight and obese college students investigated how mindfulness capability affects the association between weight‐based stigma and negative feelings (Duan & Wang, 2019). Mindfulness capability was evaluated by the Short Inventory of Mindfulness Capability (SIM‐C), which comprises three subscales (act‐awareness, describing, and non‐judging). Moreover, this study satisfied all the quality requirements. Finally, the mediation effect analysis results confirmed the indirect effects of weight stigma concern on negative emotions via mindfulness capability.

#### Academic self‐concept

Academic self‐concept is an individual's level of confidence in their own school‐based academic talents and capabilities. (Marsh & O'Neill, 1984). McGregor studied the moderator roles of academic self‐concept and social connectedness, with the latter discussed above in relation to his cross‐sectional study on male‐identifying undergraduate students (McGregor, 2022). Academic self‐concept was assessed by the Self‐Description Questionnaire III, and the quality assessment is discussed above. According to the outcomes, academic self‐concept was a significant moderator between body image and depression and therefore may be useful in preventing depressive symptoms. However, all participants were drawn from a single university, so the generalizability may be limited.

#### Comfort with weight

The study by Przezdziecki examined the mediating effect of self‐compassion, pressure from others, and comfort with weight (Przezdziecki et al., 2013). Comfort with weight was measured using a 3‐item scale, which showed that subjective evaluations of weight dissatisfaction correlate with higher levels of psychological discomfort. Along with self‐compassion, comfort with weight also met the criteria for mediation between BID and depression.

### Deteriorating mediators/moderators

#### Perceived stress and pressure

The potential influence of perceived stress was examined in two studies (Sabik et al., 2019; Ziser et al., 2019), and pressure from others was examined in one study (Przezdziecki et al., 2013). One study assessed perceived stress using the Perceived Stress Questionnaire (PSQ‐20; Ziser et al., 2019). In other research, individuals were asked to respond to stress using the Trier Social Stress Test (TSST). The Perceived Stress Scale (PSS) was used to measure participants' self‐reported felt stress, and salivary cortisol levels were recorded both before and after the TSST evaluated their biological reaction to acute stress (Sabik et al., 2019). In addition, pressure from others was assessed by four Yes/No‐format items (Przezdziecki et al., 2013). Regarding the quality assessment, confounding factors were not identified and dealt with in these studies.

Both (Ziser et al., 2019; Sabik et al., 2019) have discovered a substantial mediating role for perceived stress between BID and depression. The study on patients with obesity indicated that perceived stress mediated negative evaluation of the body and positive body dynamics and depression (Ziser et al., 2019), while another study on undergraduates discovered that chronic stress mediated the connection between body image and depressive symptoms (Sabik et al., 2019). However, pressure from others did not meet the criteria for a mediator (Przezdziecki et al., 2013). Therefore, those who feel dissatisfied with their bodies or who think that other people have a negative view of them may be more stressed and experience greater depressive symptoms.

#### Rumination

The mediation effect of rumination between BID and depressive symptoms was examined in two studies (Liu et al., 2017; Pehlivan et al., 2022). One used the Multi‐Dimensional Rumination in Illness Scale (MRIS; Pehlivan et al., 2022) and the other used the Ruminative Response Scale (RRS; Liu et al., 2017). The study of Liu et al. (2017) met all of the quality checklist requirements with flying colors, demonstrating exceptional quality, and the quality assessment of Pehlivan was mentioned in the discussion of self‐esteem. The cross‐sectional study focusing on cancer patients showed that after controlling for age, sex, marital status, education level, cancer type, cancer stage, and treatment modality, rumination mediated the relationship between body‐image concern and depression (Liu et al., 2017). However, the longitudinal study in patients with endometriosis found that rumination at T1 did not significantly mediate the body‐image and depression pathway. Poorer body image at T0 was only marginally associated with greater rumination at T1, and greater rumination at T1 was not predictive of higher depression at T2 (Pehlivan et al., 2022).

#### Parental alienation

Only one study aimed to explore the influence of parental attachment security on the relationship between BID and depression (Hoffman, 2007). The Inventory of Parent and Peer Attachment (IPPA) was used to assess parental attachment security, which included the degree of trust, communication, and alienation with respect to parents. Regarding the quality evaluation, although confounding factors were noted, the handling strategy was not made clear. The result showed that overall parental attachment security did not act as a moderator, but that parental alienation was a significant moderator. The findings suggested that the association between depressive symptoms and body dissatisfaction was exacerbated by a sense of separation from parents. The study also examined the moderating role of media exposure as measured by a daily diary, but no significant effect was found.

#### Hopelessness

Hopelessness was studied in only one cross‐sectional study, of female undergraduate students, using the Hopelessness Scale (Evans, 2011). It did not clarify the inclusion criteria and confounding factors according to the JBI quality checklist. The tenets of hopelessness depression theory were empirically supported in this study. In the structural equation model, hopelessness partially mediated the relationship between body shame and depression, which means that the association between body shame and depression resulted from the mediating role of hopelessness. Body shame significantly predicted hopelessness, which in turn predicted hopelessness depression.

#### Dysfunctional attitudes

Dysfunctional attitudes are a set of maladaptive cognitive schema that can be impacted by negative life events and can dominate and distort an individual's cognitive functions (Soo et al., 2009). A cross‐sectional study by Liu et al. investigated the mediating role of two theoretically derived intervening factors, namely dysfunctional attitudes and rumination, in adult cancer patients (Liu et al., 2017). The dysfunctional attitudes were measured by the Dysfunctional Attitudes Scale (DAS). Both factors were shown to have a significant mediating role in the relationship between BID and emotional distress.

## DISCUSSION

To our knowledge, this systematic review is the first one to comprehensively summarize the psychological mechanisms between BID and depression. In total, 23 studies were analyzed, covering various types of BID and 13 psychological variables. Furthermore, the population sample included in the analyzed research was highly diverse, including a wide range of subgroups, areas, and age categories. It is noteworthy that we included some clinical populations in which the psychological factors underlying BID and depression may differ from those in the general population owing to the disease of interest. However, because the pathogenesis of each disease is complex, further exploration is needed in the future. Meanwhile, in terms of cross‐cultural contexts there was only one original study, which found that self‐esteem had a greater mediating influence on American adolescents than on Korean adolescents (Choi & Choi, 2016). More original research is needed in the future to understand how diverse cultural backgrounds influence the effect of BID on depression. In general, our conclusion is generally tenable and relevant to a large population. Consistent evidence supports a strong mediating as well as moderating role for high self‐esteem and social support as protective factors regarding the relationship between BID and depression, whereas perceived stress as a mediator weakened.

There were seven studies that supported the significant role of self‐esteem in the relationship between BID and depression. With self‐esteem acting as a moderator, our results are in line with earlier diathesis‐stress models (Butler et al., 1994), which have suggested that having a positive self‐image is a protective factor that can reduce the influence of stressors on the incidence of depression (Gruenewald et al., 2004). Furthermore, when self‐esteem acted as a mediator, BID predicted depressive symptoms through self‐esteem, implying that body‐image dissatisfaction has the effect of reducing self‐esteem, which in turn has an influence on depressive symptoms (Duchesne et al., 2017). According to the body‐dissatisfaction‐driven hypothesis of depression, body dissatisfaction is likely to become generalized or extended to the self, and then to one's attitude to life in a self‐deprecating loop (Hartley et al., 2018). Therefore, low self‐esteem, one's overall judgment of one's worth as a person, is suggested to underlie the association between BID and depression (Rawana et al., 2010).

Together, these findings demonstrate the critical importance of having a high level of self‐esteem, and promoting self‐esteem may help reduce the negative impact of body‐related dissatisfaction on depressive symptoms in individuals. A meta‐analysis has already identified some beneficial self‐esteem‐boosting approaches, such as discussing how unique qualities set each person apart and taking part in activities that develop self‐esteem, for example making a list of positive attributes and complimenting others (Alleva et al., 2015). More research should be conducted in the future to design and assess interventions aimed at improving self‐esteem.

In addition, our review offers compelling evidence supporting both the mediating and the moderating role of social support. As a mediator, body shame can exacerbate depression by lowering an individual's social support system, which typically occurs in an anti‐fat social environment with widespread body shame. Adults concerned with their body size reported avoiding situations where they perceived they would be stigmatized (Lewis et al., 2011), thus potentially limiting their ability to feel connected. Chronic feelings of failure to meet societal norms, including those related to what constitutes an acceptable body, are highly humiliating and can lead to depression (Gilbert, 2000). When social support acts as a moderator, it is a protective factor, which may buffer the relationship between BID and depression. Social support is frequently seen as a substantial environmental resource, because the feeling of being loved, cared for, and respected by others is rationally anticipated to protect against a wide range of negative consequences (Ferreiro et al., 2012). People who are more real in their interpersonal connections report higher levels of positive body satisfaction than those who are less authentic (Gillen & Lefkowitz, 2006). Additionally, feeling more socially engaged and competent in relationships with friends and family has been linked to reduced depressive symptoms (McGregor et al., 2020). Taken together, these findings suggest that enhancing social support may help reduce depressive symptoms, particularly in people with body shame.

This review also reveals a significant mediating role of stress between BID and depression. A growing body of research has shown that threat is an important psychosocial factor contributing to stress (Dickerson & Kemeny, 2004; Townsend et al., 2011). According to social‐self‐protection theory, social‐evaluative threat occurs when an aspect of identity is at risk of being negatively appraised by others (Dickerson & Kemeny, 2004). In addition, the perception that one's appearance is negatively evaluated by others may also be associated with increased stress. Thus, individuals who are discontent with their bodies or who believe that other people may have negative opinions of their bodies may be more prone to stress. Sustained or constant stress can cause a malfunction in the stress response system, which has been linked to a number of negative physical and mental health impacts. There have been numerous studies revealing potential biological mechanisms of stress functioning. Allostatic load theory states that long‐term perceived stress can accumulate over time and affect physiological stress systems (Goldstein & McEwen, 2002). Both the hypothalamus–pituitary–adrenal (HPA) axis and the sympathetic nervous system are activated during an acute physiological stress response (Sapolsky et al., 2000), with the former resulting in the secretion of cortisol. Meanwhile, the intricate connection between depression and HPA axis activity is well described in some rich studies (Brown et al., 2004; Keller et al., 2017). Consequently, specific stress management approaches to combat body‐image dissatisfaction are required. According to one review, undertaking a thoughtful, non‐judgmental exercise in relation to one's body, such as body scanning, and reducing perceived stress may be beneficial (O'Reilly et al., 2014).

Overall, our findings corroborate the psychological pathways linking BID and depression via self‐esteem, social support, and perceived stress. Higher levels of self‐esteem and social support may attenuate the relationship between BID and depression. Although there is insufficient evidence to support other mediators and moderators, such as self‐compassion, rumination, and so on, this does not mean that these characteristics are unimportant. Future studies should explore the roles of these elements in greater depth.

Some limitations of this review should be addressed. First, the bulk of the studies included in this systematic review are cross‐sectional ones, which may overstate the moderating or mediating role of psychological variables and which do not reveal causation. Second, many included studies suffered from quality issues owing to unclear criteria for inclusion and not dealing with confounding factors, which may pose a risk for potential selection bias and confounding effects. As a result, in future studies, researchers should specify the study's inclusion and exclusion criteria in detail to increase the research quality while also providing a better foundation for future secondary research warriors. Third, the measurements of depression and BID mostly relied on self‐report and varied from study to study, making the comparison of outcomes across studies may be influenced by response bias. Currently, there are standardized and widely used scales for depression, but there is no standardized measure of BID. Future researchers are expected to produce more authoritative measures with strong reliability and validity to compensate for the current limitations.

## CONCLUSION

To summarize, the current study investigated the psychological mechanisms underlying variables such as self‐esteem, social support, and perceived stress in the association between BID and depression. Specifically, mediating and moderating roles of self‐esteem and social support as well as a mediating role of perceived stress within the association are highlighted. According to the research, interventions that focus on enhancing self‐esteem, building social support, and decreasing perceived stress may be effective in preventing and treating depression in people who are dissatisfied with their bodies.

## FUNDING INFORMATION

This work was sponsored by the National Natural Science Foundation of China (32300926) and the Ministry of Education in China Project of Humanities and Social Sciences (21YJCZH004). The funding agents had no role in the design and conduct of the study; the collection, management, and interpretation of the data; and the preparation, review, or approval of the manuscript.

## CONFLICT OF INTEREST STATEMENT

Dr. Roger S. McIntyre has received research grant support from CIHR/GACD/National Natural Science Foundation of China (NSFC) and the Milken Institute; speaker/consultation fees from Lundbeck, Janssen, Alkermes, Neumora Therapeutics, Boehringer Ingelheim, Sage, Biogen, Mitsubishi Tanabe, Purdue, Pfizer, Otsuka, Takeda, Neurocrine, Neurawell, Sunovion, Bausch Health, Axsome, Novo Nordisk, Kris, Sanofi, Eisai, Intra‐Cellular, NewBridge Pharmaceuticals, Viatris, Abbvie, Atai Life Sciences. Dr. Roger McIntyre is a CEO of Braxia Scientific Corp. The other authors declare that they have no known competing financial interests or personal relationships that could have appeared to influence the work reported in this paper.

## Supporting information


**Appendix S1.** PRISMA 2020 checklist.

**Appendix S2.** Search strategy for all the database.
**Appendix S3.** Methodological Quality and Risk of Bias Scale and Checklist Results.

## Data Availability

Data sharing is not applicable to this article as no datasets were generated or analyzed during the current study.
