# Supplementary material for: The psychological factors mediating/moderating the association between body‐image disturbance and depression: A systematic review
Source: Psych J. 2024 May 10;13(4):527–40. doi: 10.1002/pchj.754 (PMC11317192; doi:10.1002/pchj.754)
Supplement: Supplementary file 2 — Appendix S2. Search strategy for all the database. Appendix S3. Methodological Quality and Risk of Bias Scale and Checklist Results. [file PCHJ-13-527-s001.docx]

| No. | Query | Filters | Results |
| --- | --- | --- | --- |
| #1 | "depressive disorder"[MeSH Terms] OR "depression"[MeSH Terms] |  | 261,859 |
| #2 | "depressive"[Title/Abstract] OR "depress*"[Title/Abstract] OR "MDD"[Title/Abstract] OR "bipolar disorder"[Title/Abstract] |  | 605,234 |
| #3 | #1 or #2 |  | 647,947 |
| #4 | "body image disturbance"[Title/Abstract] OR "body dissatisfaction"[Title/Abstract] OR "body shame"[Title/Abstract] OR "body preoccupation"[Title/Abstract] OR "body attitudes"[Title/Abstract] OR "body image concern"[Title/Abstract] OR "body image distortion"[Title/Abstract] OR "weight concern"[Title/Abstract] |  | 4,961 |
| #5 | #3 and #4 |  | 877 |
| #6 | #3 and #4 | Meta-Analysis, Review, Systematic Review | 73 |
| #7 | #5 not #6 |  | 804 |
| #8 | #5 not #6 | Humans | 683 |

**Supplementary Appendix B** Search strategy for all the database

| No. | Query | Results |
| --- | --- | --- |
| #1 | (TS=(depression)) OR TS=(depressive disorder) and Preprint Citation Index (Exclude – Database) | 1,099,880 |
| #2 | (((TI=(depressive)) OR TI=(depress*)) OR TI=(MDD)) OR TI=(bipolar disorder) and Preprint Citation Index (Exclude – Database) | 374,544 |
| #3 | #1 or #2 | 1,147,505 |
| #4 | (((((((TI=(body image disturbance)) OR TI=(body dissatisfaction)) OR TI=(body shame)) OR TI=(body preoccupation)) OR TI=(body attitudes)) OR TI=(body image concern)) OR TI=(body image distortion)) OR TI=(weight concern) and Preprint Citation Index (Exclude – Database) | 6,296 |
| #5 | #3 and #4 and Preprint Citation Index (Exclude – Database) | 803 |
| #6 | #3 and #4 and Preprint Citation Index (Exclude – Database) and Review Article (Document Types) | 27 |
| #7 | #5 not #6 and Preprint Citation Index (Exclude – Database) | 776 |
| #8 | #5 not #6 and Preprint Citation Index (Exclude – Database) and Humans (MeSH Headings) | 401 |

Search strategy for Web of Science

Search strategy for ProQuest Central

| No. | Query | Filters | Results |
| --- | --- | --- | --- |
| S1 | mainsubject(depression) OR mainsubject(depressive disorder) |  | 326,577 |
| S2 | noft(depressive) OR noft(depress*) OR noft(MDD) OR noft(bipolar disorder) |  | 1,437,530 |
| S3 | [S1] OR [S2] |  | 1,437,537 |
| S4 | noft(body image disturbance) OR noft(body dissatisfaction) OR noft(body shame) OR noft(body preoccupation) OR noft(body attitudes) OR noft(body image concern) OR noft(body image distortion) OR noft(weight concern) |  | 109,586 |
| S5 | [S3] AND [S4] |  | 3,918 |
| S6 | [S5] | Limit to peer reviewed | 2,169 |
| S7 | [S6] | NOT (General Information AND Commentary AND Review AND Literature Review AND Correspondence AND News AND Biography AND Correction/Retraction AND Letter To The Editor AND Conference Proceeding) | 2,027 |

Search strategy for CNKI

| No. | Query | Filters | Results |
| --- | --- | --- | --- |
| #1 | 主题：抑郁症 + 抑郁症状 |  | 88,496 |
| #2 | 篇关摘：抑郁* + 重度抑郁症 + 双相情感障碍 |  | 220,244 |
| #3 | #1 or #2 |  | 251,195 |
| #4 | 主题：身体意象失调 + 身体不满 + 身材羞耻 + 身材困扰 + 身体态度 + 身体意象担忧 + 身体意象歪曲 + 体重担忧 |  | 207 |
| #5 | #3 and #4 |  | 20 |
| #6 | #3 and #4 | 综述 | 1 |
| #7 | #5 not #6 |  | 19 |

| No. | Query | Results |
| --- | --- | --- |
| #1 | [主题:(抑郁症 OR 抑郁症状)](https://webvpn.swu.edu.cn/https/537775736869676568616f78756565212ef745ba7b9b8a8fcb4228461fe3f351eece692d/advanced-search/paper?q=%E4%B8%BB%E9%A2%98:(%E6%8A%91%E9%83%81%E7%97%87%20OR%20%E6%8A%91%E9%83%81%E7%97%87%E7%8A%B6)&type=%5b) | 88,496 |
| #2 | [题名或关键词:(抑郁* OR 重度抑郁症 OR 双相情感障碍)](https://webvpn.swu.edu.cn/https/537775736869676568616f78756565212ef745ba7b9b8a8fcb4228461fe3f351eece692d/advanced-search/paper?q=%E9%A2%98%E5%90%8D%E6%88%96%E5%85%B3%E9%94%AE%E8%AF%8D:(%E6%8A%91%E9%83%81*%20OR%20%E9%87%8D%E5%BA%A6%E6%8A%91%E9%83%81%E7%97%87%20OR%20%E5%8F%8C%E7%9B%B8%E6%83%85%E6%84%9F%E9%9A%9C%E7%A2%8D)&type=%5b) | 152,819 |
| #3 | 题名或关键词:(抑郁* OR 重度抑郁症 OR 双相情感障碍) or 主题:(抑郁症 OR 抑郁症状) | 164,655 |
| #4 | 题名或关键词:(身体意象失调 OR 身体不满 OR 身材羞耻 OR 身材困扰 OR 身体态度 OR 身体意象担忧 OR 身体意象歪曲 OR 体重担忧) | 1,101 |
| #5 | 题名或关键词:(身体意象失调 OR 身体不满 OR 身材羞耻 OR 身材困扰 OR 身体态度 OR 身体意象担忧 OR 身体意象歪曲 OR 体重担忧) and 主题:(题名或关键词:(抑郁* OR 重度抑郁症 OR 双相情感障碍) or 主题:(抑郁症 OR 抑郁症状)) | 14 |

Search strategy for Wanfang

Search strategy for VIP

| No. | Query | Results |
| --- | --- | --- |
| #1 | (((((题名或关键词=抑郁症 OR 题名或关键词=抑郁症状) AND 题名或关键词=抑郁)) OR 题名或关键词=重度抑郁症) OR 题名或关键词=双相情感障碍) | 50,457 |
| #2 | (((((((题名或关键词=身体意象失调 OR 题名或关键词=身体不满) OR 题名或关键词=身材羞耻) OR 题名或关键词=身材困扰) OR 题名或关键词=身体态度) OR 题名或关键词=身体意象担忧) OR 题名或关键词=身体意象歪曲) OR 题名或关键词=体重担忧) | 104 |
| #3 | ((((((题名或关键词=抑郁) OR 题名或关键词=重度抑郁症) OR 题名或关键词=双相情感障碍) OR 题名或关键词=抑郁症) OR 题名或关键词=抑郁症状) AND (((((((题名或关键词=身体意象失调 OR 题名或关键词=身体不满) OR 题名或关键词=身材羞耻) OR 题名或关键词=身材困扰) OR 题名或关键词=身体态度) OR 题名或关键词=身体意象担忧) OR 题名或关键词=身体意象歪曲) OR 题名或关键词=体重担忧)) | 3 |

**Supplementary Appendix C** Methodological Quality and Risk of Bias Scale and Checklist Results

| Checklist Name | Author, year | Results for Each Item | | | | | | | | | Overall appraisal | | | |
| --- | --- | --- | --- | --- | --- | --- | --- | --- | --- | --- | --- | --- | --- | --- |
| JBI Critical Appraisal Checklist for Cohort Studies |  | Item 1 | Item 2 | Item 3 | Item 4 | Item 5 | Item 6 | Item 7 | Item 8 | Item 9 | | Item 10 | Item 11 |  |
|  | Brewis and Bruening, 2018 | Yes | Yes | Yes | Yes | Yes | Unclear | Yes | Yes | No | | No | Yes | Include |
|  | Ferreiro et al., 2012 | Yes | Yes | Yes | No | No | No | Yes | Yes | Yes | | No | Yes | Include |
|  | Pehlivan et al, 2022 | Yes | Yes | Yes | Yes | Yes | No | Yes | Yes | Yes | | No | Yes | Include |

| JBI Critical Appraisal Checklist for Analytical Cross-Sectional Studies |  | Item 1 | Item 2 | Item 3 | | Item 4 | | Item 5 | | Item 6 | | Item 7 | | Item 8 | |  |  |  |  | |
| --- | --- | --- | --- | --- | --- | --- | --- | --- | --- | --- | --- | --- | --- | --- | --- | --- | --- | --- | --- | --- |
|  | Brechan et al, 2015 | No | Yes | Yes | | Yes | | No | | No | | Yes | | Yes | |  |  |  | Include | |
|  | Brown, 2008 | No | Yes | Yes | | Yes | | No | | No | | Yes | | Yes | |  |  |  | Include | |
|  | Brunet et al. 2017 | No | Yes | Yes | | Yes | | Yes | | Yes | | Yes | | Yes | |  |  |  | Include | |
|  | Choi and Choi, 2016 | No | Yes | Yes | | No | | No | | No | | Yes | | Yes | |  |  |  | Include | |
|  | Duchesne et al., 2017 | No | Yes | Yes | | Yes | | Yes | | Yes | | Yes | | Yes | |  |  |  | Include | |
|  | Evans, 2011 | No | Yes | Yes | | Yes | | No | | No | | Yes | | Yes | |  |  |  | Include | |
|  | Hanley and McLaren, 2015 | No | Yes | Yes | | Yes | | Yes | | Yes | | Yes | | Yes | |  |  |  | Include | |
|  | Hassani et al, 2020 | Yes | Yes | Yes | | Yes | | No | | No | | Yes | | Yes | |  |  |  | Include | |
|  | Hoffman, 2007 | Yes | Yes | Yes | | Yes | | Yes | | Yes | | No | | Yes | |  |  |  | Include | |
|  | Koronczai et al, 2013 | No | Yes | Yes | | Yes | | No | | No | | Yes | | Yes | |  |  |  | Include | |
|  | Liu et al, 2017 | Yes | Yes | Yes | | Yes | | Yes | | Yes | | Yes | | Yes | |  |  |  | Include | |
|  | McGregor, 2021 | Yes | Yes | Yes | | Yes | | Yes | | Yes | | Yes | | Yes | |  |  |  | Include | |
|  | Morken et al, 2019 | No | Yes | Yes | | Yes | | Yes | | Yes | | Yes | | Yes | |  |  |  | Include | |
|  | Przezdziecki et al, 2013 | Yes | Yes | Yes | | Yes | | No | | No | | Yes | | Yes | |  |  |  | Include | |
|  | Roberts, 2023 | Yes | No | Yes | Yes | | Yes | | Yes | | Yes | | Yes | |  | |  |  | Include |  |
|  | Sabik et al, 2019 | Yes | Yes | Yes | Yes | | No | | No | | Yes | | Yes | |  | |  |  | Include | |
|  | Sick et al, 2020 | Yes | Yes | Yes | Yes | | Yes | | Yes | | Yes | | Yes | |  | |  |  | Include | |
|  | Todd, 2007 | No | Yes | Yes | Yes | | Yes | | Yes | | Yes | | Yes | |  | |  |  | Include | |
|  | Ziser et al, 2019 | Yes | Yes | Yes | Yes | | No | | No | | Yes | | Yes | |  | |  |  | Include | |

**JBI Critical Appraisal Checklist items for Cohort Studies**

1.Were the two groups similar and recruited from the same population?

2.Were the exposures measured similarly to assign people to both exposed and unexposed groups?

3.Was the exposure measured in a valid and reliable way?

4.Were confounding factors identified?

5.Were strategies to deal with confounding factors stated?

6.Were the groups/participants free of the outcome at the start of the study (or at the moment of exposure)?

7.Were the outcomes measured in a valid and reliable way?

8.Was the follow up time reported and sufficient to be long enough for outcomes to occur?

9.Was follow up complete, and if not, were the reasons to loss to follow up described and explored?

10.Were strategies to address incomplete follow up utilized?

11.Was appropriate statistical analysis used?

**JBI Critical Appraisal Checklist items for Analytical Cross-Sectional Studies**

1.Were the criteria for inclusion in the sample clearly defined?

2.Were the study subjects and the setting described in detail?

3.Was the exposure measured in a valid and reliable way?

4.Were objective, standard criteria used for measurement of the condition?

5.Were confounding factors identified?

6.Were strategies to deal with confounding factors stated?

7.Were the outcomes measured in a valid and reliable way?

8.Was appropriate statistical analysis used?
